# Battling COVID-19 leveraging nanobiotechnology: Gold and silver nanoparticle–B-escin conjugates as SARS-CoV-2 inhibitors

**DOI:** 10.1515/biol-2022-1047

**Published:** 2025-02-25

**Authors:** Ilyes Zatla, Lamia Boublenza

**Affiliations:** Laboratory of Microbiology Applied to the Food Industry, Biomedical and the Environment, Faculty of Natural and Life Sciences, Earth and Universe Sciences, Department of Biology, University of Abou Bekr Belkaid, Tlemcen, 13000, Algeria

**Keywords:** SARS-CoV-2, COVID-19, antivirals, nanoparticles, biomolecules, molecular dynamics

## Abstract

The COVID-19 pandemic, an unprecedented global health crisis, has thrust humanity into a relentless battle with a variety of treatments and vaccines against the SARS-CoV-2 virus. Recent developments in nanotechnology have garnered significant interest in the application of metallic nanoparticles (NPs); specifically, silver nanoparticles (AgNPs) and gold nanoparticles (AuNPs) have demonstrated antimicrobial and antiviral properties. This study investigates the molecular interactions between the receptor binding domains of five SARS-CoV-2 spike protein variants (Alpha, Beta, Delta, Omicron, and Gamma) and the angiotensin-converting enzyme 2 (ACE2) receptor, followed by the docking of AuNPs and AgNPs and the natural compound Beta-escin onto these complexes. As well as the inspection of both NPs against the virus main protease (Mpro) and RNA-dependent RNA polymerase (RdRp). Comprehensive computational simulations utilizing Autodock 4.2 and HDOCK server were employed to evaluate the binding affinities of these NPs toward key viral targets, SARS-CoV-2 Mpro, RdRp, and the spike glycoprotein. The results revealed that both AgNPs and AuNPs exhibited successful binding to the active pockets of SARS-CoV-2 Mpro, with slightly varying binding energies. In contrast, for RdRp, AgNPs demonstrated superior binding affinity compared to AuNPs, with differences in the residues involved in the binding pocket. AuNPs exhibited stronger binding affinities in the spike protein pocket. We also determined robust binding affinities between ACE2 and the spike variants, with the Omicron variant exhibiting the highest affinity. Subsequent docking of AuNPs and AgNPs revealed strong interactions with all ACE2–spike complexes, with AuNPs showing slightly higher affinities. The findings contribute to a deeper understanding of the interactions between NPs and viral proteins, shedding light on their mechanisms of action and their potential to offer innovative solutions for combating infectious diseases, particularly those caused by SARS-CoV-2.

## 1 Introduction

The COVID-19 pandemic, caused by the SARS-CoV-2 virus, has precipitated a global health crisis, necessitating the rapid development of effective therapeutic and diagnostic strategies [1]. Central to the virus’s pathogenicity is the spike protein, which facilitates viral entry by binding to the angiotensin-converting enzyme 2 (ACE2) receptor on host cells. Variants of concern such as Alpha, Beta, Delta, Omicron, and Gamma have emerged, characterized by mutations in the spike protein that enhance transmissibility and potentially alter vaccine efficacy [2]. Understanding the molecular interactions between these spike protein variants and the ACE2 receptor is critical for developing targeted interventions. Molecular docking, a computational method that predicts the preferred orientation of one molecule to a second, provides valuable insights into these interactions [3]. Also, among the various targets within the realm of coronaviruses, particular focus has been placed on the main protease (Mpro), RNA-dependent RNA polymerase (RDRP), and the spike protein [4].

These metallic nanoparticles (NPs), notably silver and gold, have emerged as highly promising agents due to their demonstrated effectiveness against a spectrum of microorganisms, including bacteria, viruses, and other eukaryotic entities. This heightened interest among researchers is a response to the escalating problem of microbial resistance and the development of resilient strains [5,6]. Silver nanoparticles (AgNPs) exhibit remarkable antimicrobial properties attributed to their distinctive physiochemical attributes and superior biological functionalities [7]. They also possess antiviral properties, with reports indicating that AgNPs synthesized in Hepes buffer can impede the replication of HIV-1, displaying anti-human immunodeficiency virus (HIV) activity, in addition to inhibiting hepatitis B virus and herpes simplex virus [8,9]. Gold nanoparticles (AuNPs) have garnered significant attention in various domains, including industry and nanomedicine, owing to their exceptional electrical, optical, mechanical, and biological attributes [10,11]. The antiviral properties of AuNPs have been supported by research studies, with their minute particle size, low toxicity, non-immunogenicity, and capacity to bind biological ligands rendering them promising candidates for antibacterial and antiviral purposes. Previous investigations have demonstrated the ability of AuNPs to hinder multiple stages in the life cycles of various viruses, including Herpes simplex virus type 1, Zika virus, HIV, dengue virus, and foot-and-mouth disease virus [12,13]. Beta-escin (B-escin) is a natural saponin compound extracted from the seeds of the horse chestnut tree (*Aesculus hippocastanum*). Known for its anti-inflammatory, antiviral, vasoprotective, and anti-edematous properties, it has been used in traditional medicine and continues to be explored for its therapeutic potential [14,15,16]. Investigating its synergistic potential when combined with NPs in binding to the ACE2–spike complex could reveal novel strategies to combat SARS-CoV-2 [17].

The primary objective of this study is to investigate and elucidate the binding affinities and interaction dynamics among the molecules including the ACE2 receptor and the five SARS-CoV-2 spike protein variants, with the Au and Ag NPs onto these protein–protein complexes, followed by B-escin. And also to explore the potential of AgNPs and AuNPs interactions with key viral targets, namely the Mpro and RdRp, providing insights into their potential therapeutic and diagnostic applications in managing COVID-19 and its variants.

## 2 Materials and methods

The molecular docking procedure involved utilizing the HDOCK server to conduct docking simulations for the receptor binding domain (RBD) of five different SARS-CoV-2 spike protein variants: Alpha, Beta, Delta, Omicron, and Gamma (Protein Data Bank [PDB] ID 7R13, 7WEV, 7VHH, 7QO7, and 8DLO, respectively). In this investigation, the amino acid sequence of ACE2 wild type (PDB ID 1R42) served as the ligand, while the SARS-CoV-2 spike protein RBD variants acted as receptors. Subsequently, AuNPs and AgNPs were docked onto the resulting protein–protein complexes. The structural information for AgNPs (CID_23954) and AuNPs (CID_23985) was retrieved from PubChem, and their geometries underwent optimization through Autodock 4.2 [18,19]. Crystal structures’ water molecules were removed, polar hydrogen atoms were added, and only the relevant chains were retained. Marsili–Gasteiger partial charges were assigned using a two-phase algorithm. The receptor crystal structures underwent energy minimization via the AutoDockTools (ADT, v1.5.6) prepare_receptor4.py command, employing Kollman-united charges for calculating partial atomic charges [20]. For NP docking, it was allowed to be docked in all the protein pockets to find a suitable site for binding.

B-escin structure was allowed to dock against all the 10 obtained Au/Ag NP–spike–ACE2 complexes. The docking parameters were set to an energy evaluation of 2500.00, 100 runs, and a population size of 150, based on the Lamarckian genetic algorithm [21]. Ten binding modes were produced for the ligand with a maximum of 3 kcal/mol energy difference between each mode, and the best conformations, showing the lowest binding free energy, were retrieved. PyMOL(TM) 2.5.2 was used for generating 3D interaction figures [22].

Both AgNPs and AuNPs were tested again against two crystal structures of key proteins of SARS-CoV-2 that were chosen and procured from the Protein Data Bank M^pro^ (PDB ID 7CAM), RdRp (PDB ID 6YYT), which were all obtained from Zhang Lab’s I-Tasser receptor-binding domain, to find out a potent target for of the NPs [23,24].

In preparing the protein for the molecular docking, only the needed chains were kept, and the receptor structures were prepared using the AutoDock tool. Water molecules in the three crystal structures were deleted, while polar hydrogen atoms were added. Marsili–Gasteiger partial charges were appointed employing a two-phase algorithm. Furthermore, the receptor crystal structures were subjected to energy minimization using the AutoDockTools 1.5.6 applying the command, prepare_receptor4.py where Kollman-united charge was used for calculating the partial atomic charge. In determining the preferred binding sites of the protein, the grid box center *xyz*-coordinates were set to 13.688, 13.076, 5.396 for M^pro^, 89.178, 106.82, and 99.62 for RdRp. The outputs from AutoDock were further analyzed through the UCSF Chimera and PyMOL software package [25].

## 3 Results and discussion

The molecular docking studies conducted using the HDOCK server reveal significant insights into the binding interactions between ACE2 and various spike protein variants of the SARS-CoV-2 virus. The results in Table 1 underscore a strong affinity of ACE2 toward all examined spike receptor variants, with docking scores ranging from −269.59 to −359.33 kcal/mol. Notably, the Omicron variant exhibited the highest binding affinity, succeeded by the Delta variant, while the Gamma variant displayed the least binding affinity. These findings align with the epidemiological data suggesting higher transmissibility associated with the Omicron variant, potentially due to its robust interaction with ACE2 [26,27,28].

**Table 1:** HDOCK results of docking ACE2 as a ligand on the five spike receptor variants

Complex	ACE2–spike Alpha variant	ACE2–spike Beta variant	ACE2–spike Delta variant	ACE2–spike Omicron variant	ACE2–spike Gamma variant
Docking score (kcal/mol)	−298.59	−281.90	−333.36	−359.33	−269.59
Confidence score	0.9513	0.9333	0.9751	0.9850	0.9162
Ligand rmsd (Å)	1.19	84.66	0.80	0.88	47.17

Corroborating the docking scores, the confidence score, reflecting the probability of binding between two molecules, showed a consistent correlation. The ACE2–spike Omicron variant recorded the highest confidence score (0.9850), followed closely by the ACE2–spike Beta variant (0.9751). Conversely, the ACE2–spike Gamma variant reported the lowest confidence score at 0.9162. Given that a confidence score exceeding 0.7 indicates a high probability of binding, these scores affirm that all five tested interactions possess a strong binding capability. These data provide a molecular basis for the varying degrees of infectivity observed with different variants, highlighting the enhanced binding efficiency of the Omicron variant as a potential factor in its rapid spread.

The docking analysis of AuNPs and AgNPs onto the resulting protein–protein complexes, as detailed in Table 2, underscores their notable affinity toward all five ACE2–spike receptor variants. Notably, the binding affinities range between 0.18 and 0.27 kcal/mol, indicative of strong interactions. Across the complexes, AuNPs generally exhibit a slightly higher affinity compared to AgNPs. Particularly noteworthy is the elevated affinity observed towards the ACE2–spike Beta variant, while the ACE2–spike Omicron variant shows relatively lower affinity. Detailed interactions are provided in Table 3, highlighting the 2D interactions of both AgNPs and AuNPs across all ACE2–spike receptor variant complexes. Remarkably, both NPs demonstrate consistent binding within the same pocket across all variants, emphasizing their consistent mode of interaction.

**Table 2:** Binding energy results of docking AuNPs and AgNPs on ACE2–5 spike receptor variant complexes

Ligand	Score	ACE2–spike Alpha variant	ACE2–spike Beta variant	ACE2–spike Delta variant	ACE2–spike Omicron variant	ACE2–spike Gamma variant
AgNPs	Binding energy (kcal/mol)	−0.21	−0.23	−0.22	−0.18	−0.20
AuNPs	Binding energy (kcal/mol)	−0.25	−0.27	−0.26	−0.20	−0.23

**Table 3:** 2D interaction results from docking AuNPs and AgNPs on ACE2–5 spike receptor variant complexes

Ligand	ACE2–spike Alpha variant	ACE2–spike Beta variant	ACE2–spike Delta variant	ACE2–spike Omicron variant	ACE2–spike Gamma variant
AgNPs	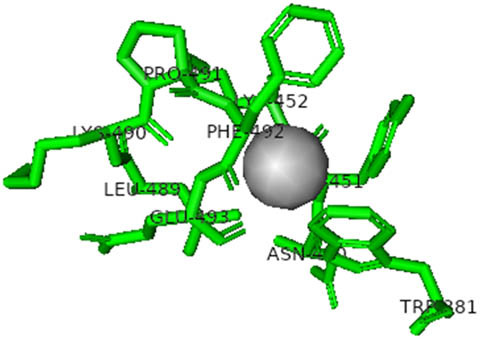	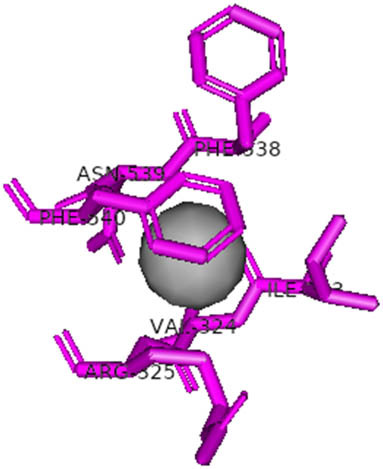	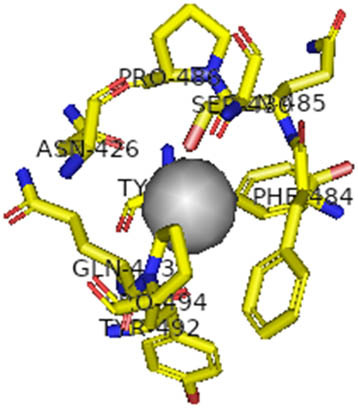	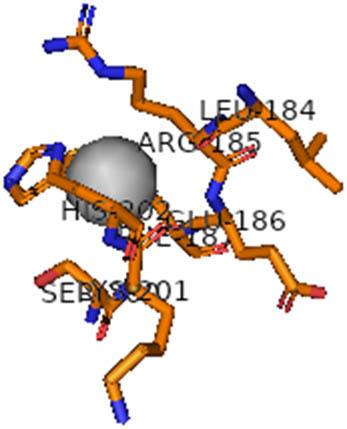	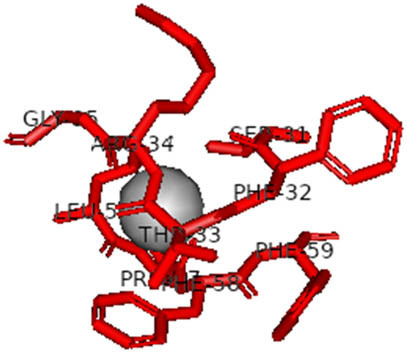
AuNPs	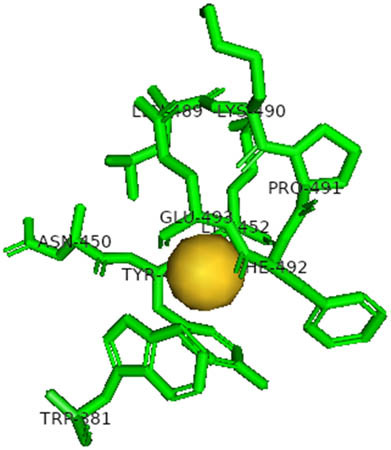	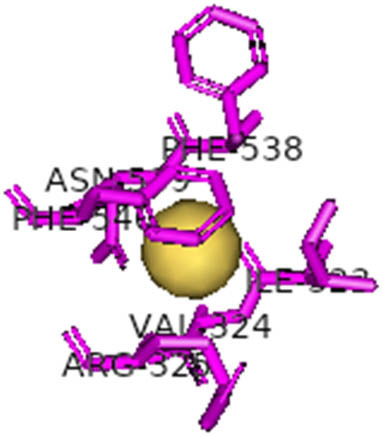	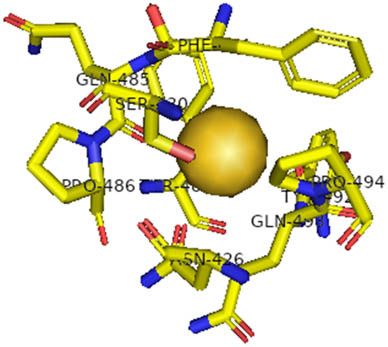	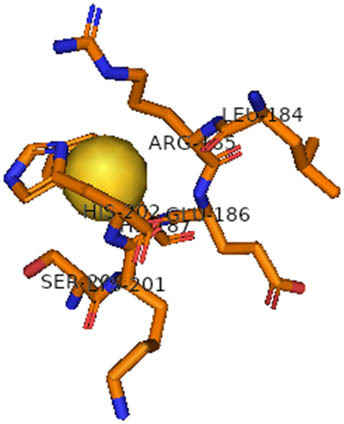	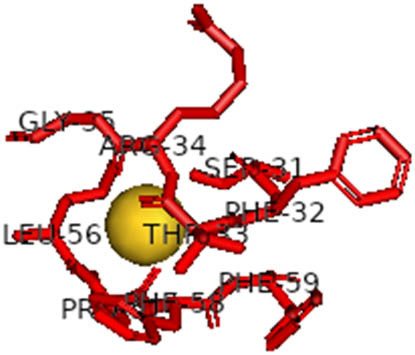

Our docking results indicate strong interactions between AuNPs/AgNPs and the active sites of Mpro and RdRp. Mpro, being essential for processing viral polyproteins, plays a pivotal role in viral replication. Inhibiting Mpro’s active site may prevent the proteolytic cleavage necessary for generating functional non-structural proteins, ultimately halting viral replication. Similarly, RdRp is critical for synthesizing viral RNA, and its inhibition would disrupt the transcription and replication of the viral genome.

The strong binding affinities observed in this study suggest that AuNPs/AgNPs may act as steric inhibitors, blocking substrate access or inducing conformational changes that impair enzymatic activity. Additionally, the unique properties of metallic NPs, such as their ability to generate reactive oxygen species (ROS) and disrupt viral protein structures, may further enhance their antiviral efficacy. However, further experimental validation is necessary to confirm these proposed mechanisms.

The resulting docked complexes of NPs were further examined through docking with B-escin to evaluate potential synergistic activity. B-escin’s amphipathic nature also allows it to integrate into lipid membranes, which could potentially destabilize the viral envelope, further impairing infectivity. Additionally, B-escin’s ability to modulate host immune responses by reducing inflammation and oxidative stress may provide a dual benefit in combating the virus and mitigating COVID-19-associated complications. However, further experimental and pharmacological studies are required to validate these findings and establish their efficacy and safety *in vivo*. As presented in Table 4, B-escin exhibited notable binding affinities across the 10 NP–ACE2–spike variant complexes, ranging from −8.11 to −10.46. Notably, the highest affinity was observed toward the NP–ACE2–spike Alpha variant, while the lowest was toward the NP–ACE2–spike Delta variant. In each complex, B-escin occupied distinct binding pockets, as delineated in Table 5.

**Table 4:** Docking binding energy results of B-escin docked on AuNP and AgNP complexes with ACE2–5 spike receptor variant complexes

Ligand	NP –complex	ACE2–spike Alpha variant	ACE2–spike Beta variant	ACE2–spike Delta variant	ACE2–spike Omicron variant	ACE2–spike Gamma variant
B-escin	AgNPs	−10.46	−9.22	−8.50	−8.98	−9.29
	AuNPs	−10.21	−9.35	−8.11	−9.25	−8.94

**Table 5:** 2D interaction results from docking B-escin on NPs–ACE2–spike receptor variant complexes

B-escin–NP complex	ACE2–spike Alpha variant	ACE2–spike Beta variant	ACE2–spike Delta variant
AgNPs	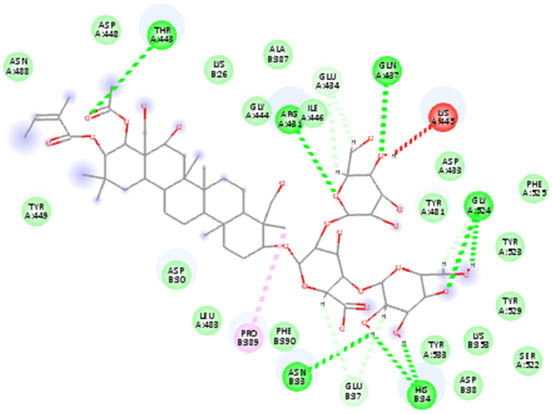	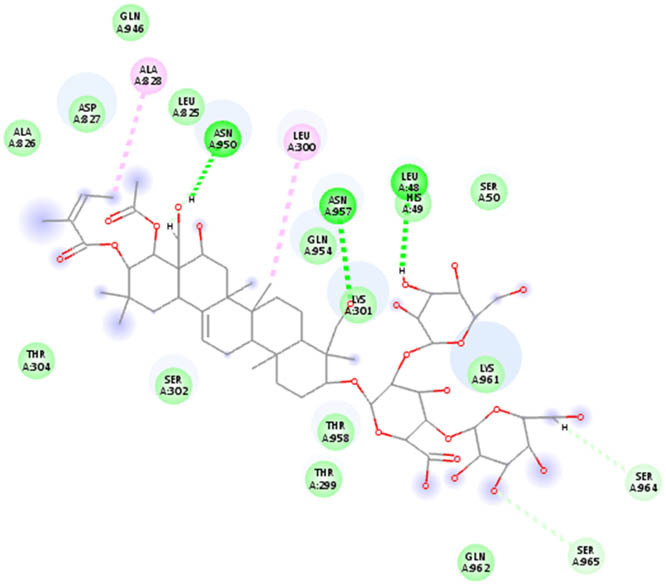	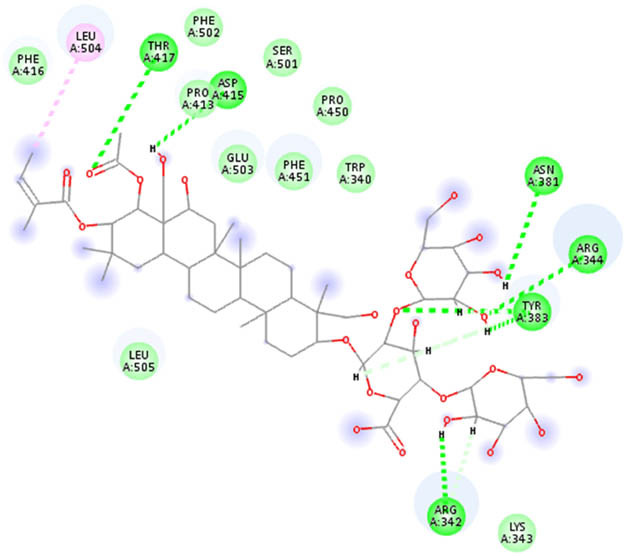
AuNPs	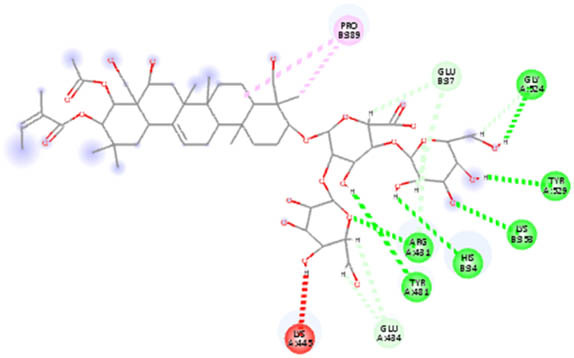	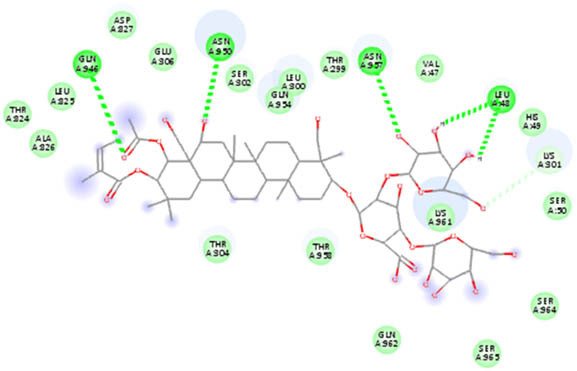	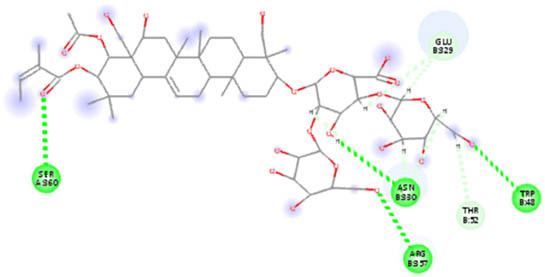

B-escin–NP complex	ACE2–spike Omicron variant	ACE2–spike Gamma variant
AgNPs	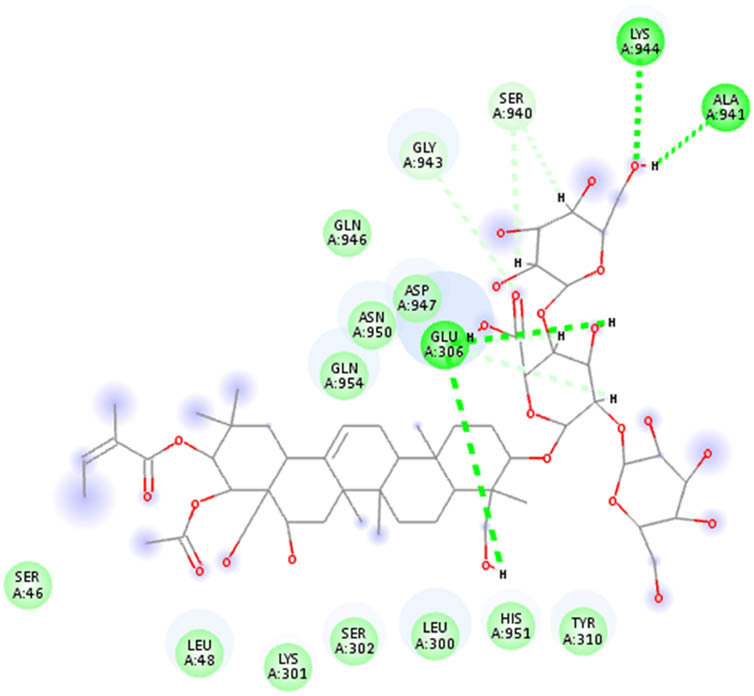	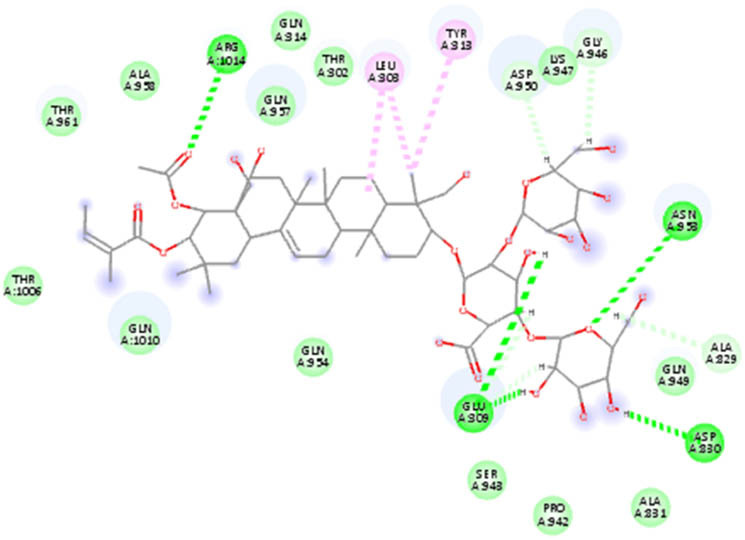
AuNPs	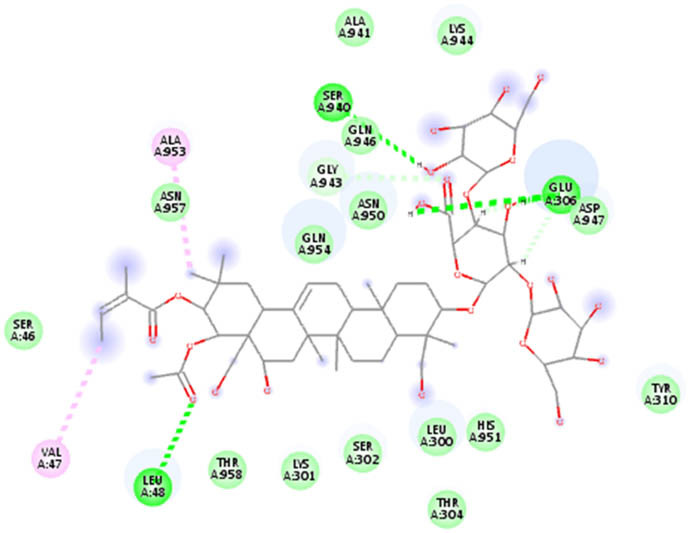	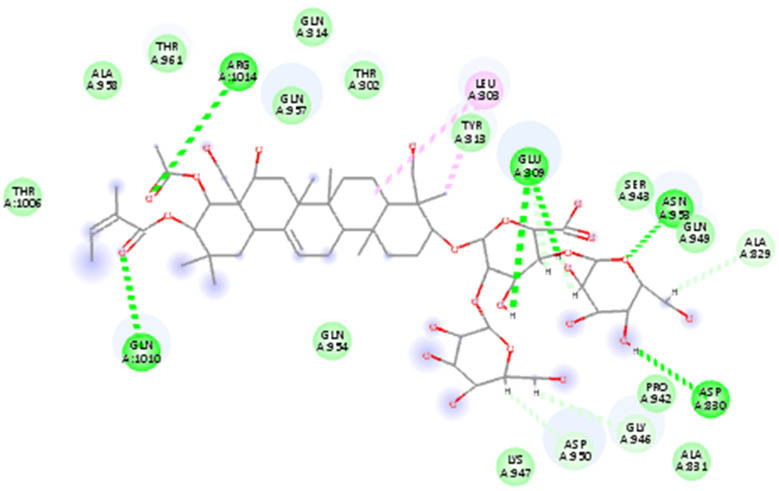

In the NP–ACE2–spike Alpha variant complexes of both Ag and AuNPs, B-escin preferentially bound to a pocket at the intersection of ACE2 and spike proteins, forming eight and six hydrogen bonds, respectively, with the AgNP and AuNP complexes (Table 5). Despite the numerous interactions and high binding scores (−10.46 and −10.21 kcal/mol for AgNP and AuNP complexes, respectively), some unfavorable interactions were noted in both complexes.

For the NP–ACE2–spike Beta variant complexes of Ag and AuNPs, B-escin bound to a pocket at the center of the spike beta variant, demonstrating significant binding affinities (−9.22 and −9.35, respectively) (Table 4). In these complexes, B-escin formed three and five hydrogen bonds, respectively, with no unfavorable clashes observed, unlike the alpha variants.

Distinct from the alpha and beta complexes, B-escin adopted two different binding pockets when docked onto Ag and AuNP–ACE2–spike Delta variant complexes. It occupied a terminal pocket in the AgNP–ACE2–spike Delta variant complex and a pocket at the intersection of both proteins in the AuNP–ACE2–spike Delta variant complex. Although the binding affinities toward Ag and AuNP–ACE2–spike Delta variant complexes were the lowest (−8.5 and −8.11, respectively) (Table 4), numerous hydrogen bond interactions were formed with their binding pockets (6 and 4, respectively) (Table 5).

In both NP–ACE2–spike Omicron and Gamma variant complexes, B-escin exhibited similar binding affinities (Table 4) with a large number of favorable binding interactions (Table 5). It predominantly occupied pockets at the center of the spike variant protein, akin to the reported interactions with NP–ACE2–spike Beta variant complexes.

This study also investigated the potency of AgNPs and AuNPs against the M^pro^ and the RdRp. The choice of these proteins lies in the insightful features and key roles they play in the virus. In particular, primary proteases (Mpros), also known as 3-chymotrypsin-like proteases (3CLpros), are a group of cysteine hydrolases found in β-coronaviruses that are highly conserved. Growing evidence suggests that these 3CLpro enzymes are vital for viral replication and are crucial targets for the prevention and treatment of infectious diseases caused by coronaviruses, such as COVID-19. RNA-dependent RNA polymerase (RdRp) is involved in both RNA replication and transcription, facilitating the production of an RNA strand that is complementary to a specific RNA template through catalysis. This protein is highly considered because it is employed by SARS-CoV-2 to replicate its genetic material and transcribe its genetic information. The result of this analysis disclosed that both AgNPs and AuNPs were able to fit into the active pockets of SARS-CoV-2 M^pro^ with binding energies of −0.16 and −0.13 kcal/mol, respectively. Their 3D interactions (Table 6) showed that they occupied the same pocket as reported in the literature, and the residues involved in this binding affinity are listed in Table 6. His41, an important residue involved in interactions with potential inhibitors, as reported in the literature, is involved in both pockets [29]. Specifically, upon targeting the AgNPs and AuNPs on the primary protease, binding affinities of −0.16 and −0.13 kcal/mol, respectively, were reported. The residues associated with the interaction with AgNPs are Pro39, Arg40, His41, Tyr45, Cys85, His164, Phe181, and Asp187. The Pro39, Arg40, His41, Cys85, and Asp187 residues were shown to interact with AuNPs. When the AgNP and AuNp were targeted at RdRp, binding affinities of −0.13 and −0.07 kcal/mol, respectively, were recorded. These interactions were observed with Gly485, Gly486, His572, Gln573, Lys574, and Leu575 for AgNPs and with Ser564, Thr565, Asn568, Asp684, and Ala685 for AuNPs. Further exploration of the effects of the compounds against protein spikes, −0.13 and −0.17 kcal/mol, was reported for AgNPs and AuNPs, respectively. This interaction is observed with the Asn121, Asn122, Ala123, Thr124, Phe 175, and Leu 176 residues of AgNPs and with the Asn121, Asn122, Ala123, Thr124, Asn125, Val126, Phe175, Leu176, and Met177 residues of AuNPs. In comparison, AgNPs show better binding affinity than AuNPs when targeted at the RdRp. The residues involved in the pocket are completely different from those of the NPs, as presented in Table 6. On the other hand, AuNPs showed better binding affinities for spike pockets than AgNPs, as more pocket residues were involved in this interaction. The identified residues involved in this interaction are the same as those reported for other reported inhibitors, such as elbasvir (DB11574), daclatasvir (DB09102), and glecaprevir (DB13879) [30].

**Table 6:** PDB ID targets and bonded residues’ 3D interactions with their receptors and their binding energies

PDB	NP	Binding energy (kcal/mol)	Residues	3D interactions
PDB ID 7CAM (M^pro^)	AgNP	−0.16	Pro39, Arg40 His41, Tyr45, Cys85, His164, Phe181, Asp187	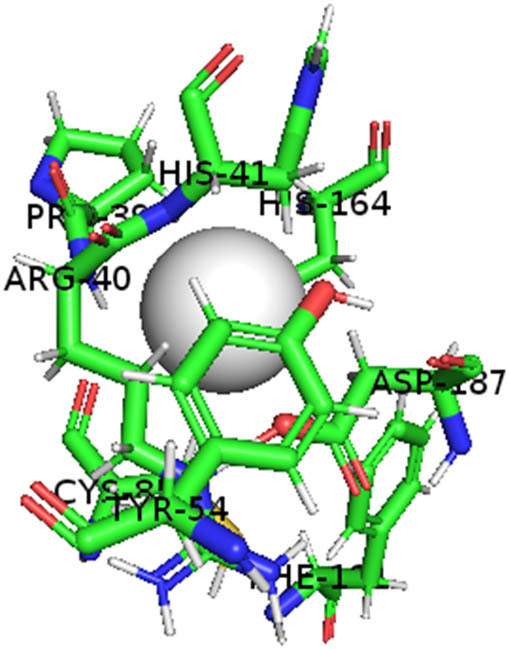
	AuNP	−0.13	Pro39, Arg40, His41, Cys85, Asp187	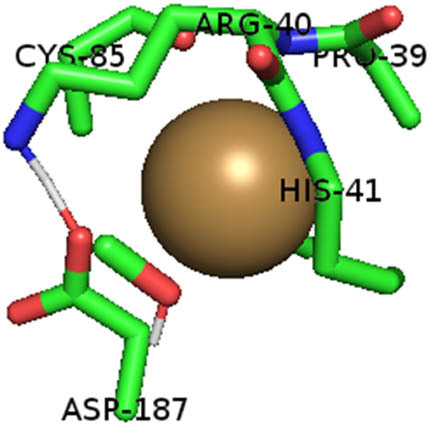
PDB ID 6YYT (RdRp)	AgNP	−0.13	Gly485, Gly486, His572, Gln573, Lys574, Leu575	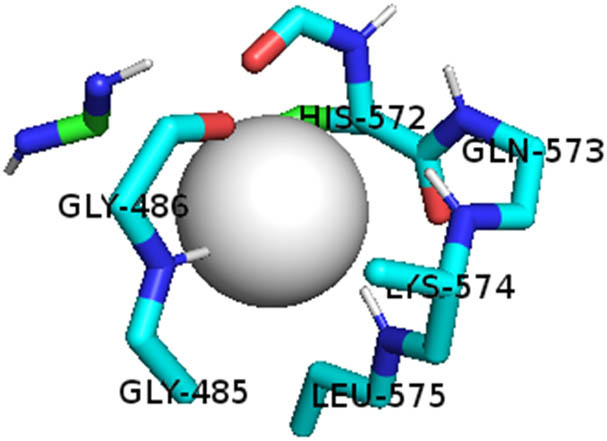
	AuNP	−0.07	Ser564, Thr565, Asn568, Asp684, Ala685	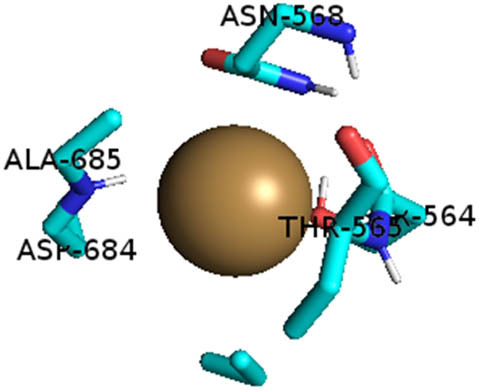
PDB ID 6VXX (spike)	AgNP	−0.14	Asn121, Asn122, Ala123, Thr124, Phe175, Leu176	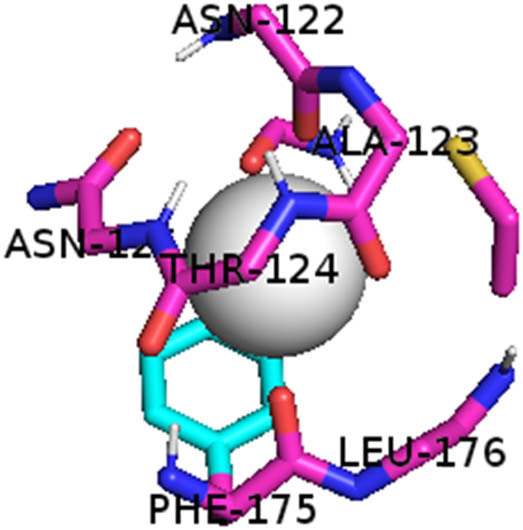
	AuNP	−0.17	Asn121, Asn122, Ala123, Thr124, Asn125, Val126, Phe175, Leu176, Met177	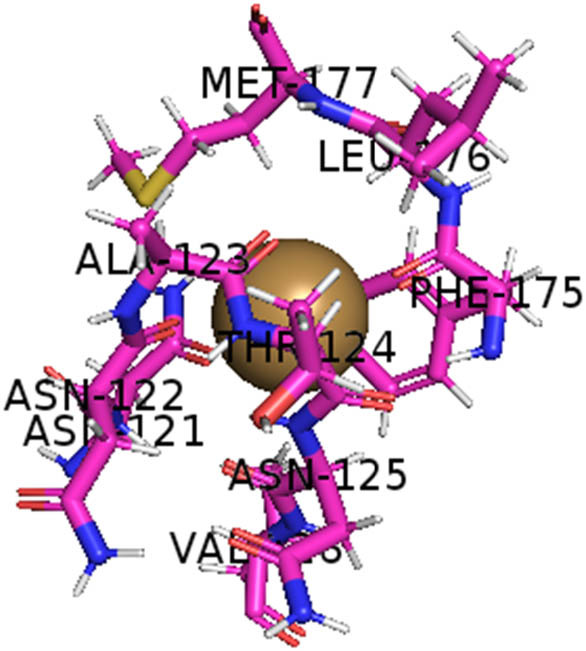

While this study provides valuable insights into the binding interactions between SARS-CoV-2 spike protein variants, ACE2, NPs, and B-escin, it is important to acknowledge several limitations. First, the results are derived solely from *in silico* molecular docking simulations, which may not fully replicate dynamic biological systems. Experimental validation, including *in vitro* and *in vivo* assays, is essential to confirm these findings. Second, this study does not evaluate the cytotoxicity or biocompatibility of AuNPs and AgNPs, which are critical considerations for therapeutic applications.

NPs, while promising, are known to exhibit dose-dependent toxicity. For example, AgNPs can induce oxidative stress by generating ROS, potentially leading to cellular damage. Furthermore, NP accumulation in vital organs, such as the liver and kidneys, has been observed in preclinical studies, raising concerns about their long-term safety. Mitigation strategies, such as NP functionalization with biocompatible materials and rigorous toxicity testing, are imperative before considering clinical applications. These limitations underline the necessity for a cautious approach and further experimental studies to assess the safety and efficacy of these therapeutic agents.

While B-escin has demonstrated promising pharmacological activity, it is not without limitations. Potential side effects include gastrointestinal disturbances such as nausea, diarrhea, and, in rare cases, allergic reactions. Additionally, caution is advised in patients with renal impairment due to potential nephrotoxic effects at higher doses. Regarding the spike protein, it is important to emphasize that its use in this study is limited to *in silico* simulations using structural data from the PDB. In therapeutic applications, proteins or their derivatives undergo extensive modifications to ensure safety and efficacy. Further preclinical studies are required to assess the safety, bioavailability, and therapeutic window of B-escin and NP-based delivery systems before advancing to clinical trials. These steps are crucial to ensure the safety of potential pharmacological interventions.

## 4 Conclusion

In summary, the docking analysis highlights the robust affinity of AuNPs and AgNPs toward various ACE2–spike receptor variants. Furthermore, the distinct binding pockets and interactions observed underscore the nuanced nature of NP–protein interactions. These findings not only deepen our understanding of NP–receptor interactions but also underscore their potential as therapeutic agents or diagnostic tools against viral infections. The docking study elucidates the synergistic potential of B-escin with both AuNPs and AgNPs across various ACE2–spike receptor variant complexes. The varying binding affinities and preferred binding pockets of B-escin underscore its dynamic interaction with NP–protein complexes. Despite differences in binding preferences, B-escin demonstrates consistently favorable interactions, suggesting its potential as a therapeutic adjunct in combating viral infections mediated by ACE2–spike receptor variants. Both AgNPs and AuNPs have also shown binding affinities to the Mpro and RDRP, suggesting their potential in antiviral drug development. These insights pave the way for further exploration of B-escin–NP combinations as potential antiviral strategies.
